# Local infiltration analgesia: a 2-year follow-up of patients undergoing total hip arthroplasty

**DOI:** 10.1007/s00540-017-2403-6

**Published:** 2017-08-30

**Authors:** Ján Kuchálik, Anders Magnuson, Anders Lundin, Anil Gupta

**Affiliations:** 1 Department of Anaesthesiology and Intensive Care, Institution for Medicine and Health, Örebro University Hospital, Örebro, Sweden; 2 Department of Orthopaedic Surgery, Institution for Medicine and Health, Örebro University Hospital, Örebro, Sweden; 3 Clinical Epidemiology and Biostatistics Unit, Örebro University Hospital, Örebro, Sweden; 4 Department of Anaesthesiology and Intensive Care, Karolinska University Hospital Solna–Karolinska Institutet, Stockholm, 17176 Sweden

**Keywords:** Local anesthetics, Quality of life, Total hip arthroplasty, Postoperative complications

## Abstract

**Purpose:**

Local infiltration analgesia (LIA) is commonly used for postoperative pain management following total hip arthroplasty (THA). However, the long-term effects of the component drugs are unclear. The aim of our study was to investigate functional outcome, quality of life, chronic post-surgical pain, and adverse events in patients within 2 years of undergoing THA.

**Methods:**

The study was a secondary analysis of data from a previous larger study. Eighty patients were randomized to receive either intrathecal morphine (Group ITM) or local infiltration analgesia (Group LIA) for pain management in a double-blind study. The parameters measured were patient-assessed functional outcome [using the Hip dysfunction and Osteo-arthritis Outcome Score (HOOS) questionnaire], health-related quality of life [using the European Quality of Life–5 dimensions (EQ-5D) questionnaire and the 36-Item Short Form Health Survey (SF-36) score], and pain using the numeric rating score (NRS), with persistent post-surgical pain having a NRS of > 3 or a HOOS pain sub-score of > 30. All complications and adverse events were investigated during the first 2 years after primary surgery.

**Results:**

Pain intensity and rescue analgesic consumption were similar between the groups after hospital discharge. No differences were found in HOOS or SF-36 score between the groups up to 6 months after surgery. A significant group × time interaction was seen in the EQ 5D form in favor of the LIA group. No between-group difference in persistent post-surgical pain was found at 3 or 6 months, or in adverse events up to 2 years after surgery.

**Conclusion:**

Analysis of functional outcome, quality of life, and post-discharge surgical pain did not reveal significant differences between patients receiving LIA and those receiving ITM. LIA was found to be a safe technique for THA during the long-term follow-up. However, it should be noted that these conclusions are based on a limited number of patients.

## Introduction

Total hip arthroplasty (THA) is a common and standardized surgical procedure. Postoperative mortality after hip joint replacement is low, but a number of complications remain, including persistent post-surgical pain (PPSP) [1], hip dislocation [2], infection [3], and deep vein thrombosis [4]. Strategies to reduce morbidity and mortality include the posterior surgical approach, mechanical and pharmacological prophylaxis of deep vein thrombosis, and the use of spinal anesthesia [5]. One of the important factors affecting patient satisfaction with THA is good postoperative pain management [6]. Poorly managed postoperative pain can lead to chronic post-surgical pain and, therefore, aggressive postoperative pain management is important [7]. Local infiltration analgesia (LIA) using a combination of large-volume local anesthetics (LA) and non-steroidal anti-inflammatory drugs (NSAIDs) injected systematically peri-articularly has been used for pain management with variable success [8]. In two earlier studies, we found improved analgesia when using LIA as compared to intrathecal morphine (ITM) during the first few days postoperatively after knee and hip arthroplasty [9, 10].

The use of NSAIDs administered orally or systemically during orthopedic surgery has been controversial due to the risk of delayed healing from the inhibition of osteoclast and osteoblast formation [11], specifically during fracture surgery. LA injected intra-articularly have also been suspected to cause chondroplasia in the presence of intact cartilage [12]. Thus, delayed healing and functional recovery, local anesthetic toxicity, PPSP, and infection may be some of the risks associated with the use of LIA. We hypothesized that LIA has no negative effects on post-discharge and chronic post-surgical pain, mobility, recovery of function, quality of life, or major postoperative complications. Therefore, the primary aim of our study was to assess hip function and the secondary aims were to assess quality of life, post-discharge and PPSP at 6 months after surgery and all major complications at up to 2 years after surgery.

## Methods

Approval for this study was obtained from the Regional Ethics committee in Uppsala as well as from the Swedish agency on drugs prior to patient recruitment for the initial larger study. The initial larger study was registered in Clinicaltrials.gov (Identification number: NCT01281891) and monitored by an independent organization based in the hospital for quality control; it was conducted in accordance with Good Clinical Practice. Patients were recruited from the Department of Orthopedic Surgery, University Hospital, Örebro, Sweden between 2011 and 2012. The study reported here is a secondary analysis of the data from the initial study on post-discharge events, including hip function and quality of life. These data are therefore part of the larger study but have never been published; in contrast, the primary data on early postoperative recovery and postoperative pain management have been published previously [10].

A total of 80 patients in the age group 50–85 years, with ASA physical status I–II, who were undergoing elective THA were randomized in this prospective, double blind, parallel group, longitudinal study. Written and verbal informed consent was obtained from all patients prior to inclusion. The exclusion criteria were chronic pain requiring opioid medication, known allergy to local anesthetics, contraindications for using NSAIDs or receiving regional anesthesia, and inability to follow verbal or written instructions. Patients were allocated to one of two groups, namely, Group ITM (standard of care at our hospital) and Group LIA, according to a computer-generated randomization sequence by the hospital pharmacy. Thus, double-blinded syringes were sent to the operating room with the drugs/placebo according to the randomization sequence.

### Anesthesia and surgery

Detailed information was provided to all patients on the surgery, anesthesia, postoperative pain management, and physiotherapy and all patients were asked to complete several questionnaires relating to hip function, pain and health-related quality of life (see section Recordings and measurements). Additionally, a preoperative pain score was obtained using a numeric rating scale (NRS) where 0 = no pain and 10 = worst imaginable pain. All patients were injected subcutaneously once daily with dalteparin 5000 IU as prophylaxis against deep vein thrombosis for 10 days.

Anesthesia and surgery were standardized. All patients received spinal anesthesia with bupivacaine 17.5 mg and either an additional intrathecal administration of 0.1 mg morphine (Group ITM) or an equal volume of saline (Group LIA). In Group LIA, 151.5 ml of a mixture consisting of a long-acting local anesthetic (ropivacaine 2 mg/ml = 150 ml), a NSAID (ketorolac 30 mg/ml = 1 ml), and epinephrine (1 mg/ml = 0.5 ml) was injected into the periarticular tissues in a systematic way as described previously; in Group ITM, an equivalent amount of saline was injected. Propofol was administered intravenously during surgery for sedation, if needed. At the end of surgery, a multi-hole catheter was tunneled about 8–10 cm from the skin incision and placed intra-articularly and connected to a bacterial filter under sterile conditions. After 24 h, 22 ml of active drugs (ropivacaine 0.2%, 20 ml; ketorolac 30 mg, 1 ml; adrenaline 0.1 mg, 1 ml) was injected in Group LIA patients via the catheter, and an equal amount of saline was injected into Group ITM patients, after which the catheter was withdrawn. Tramadol and paracetamol were administered as needed as rescue medication following surgery and home discharge. Cloxacillin 1 g was given intravenously before surgery and at 8, 16, and 24 h postoperatively.

### Recordings and measurements

- Pain (NRS). Pain now and average pain over the previous week was evaluated at then end of postoperative weeks 1, 2, 3, and 4 using a questionnaire. At 3 and 6 months pain intensity was recorded as pain on movement (walking or standing) when performing activities of daily living and was determined by a telephone call.
- Analgesic consumption. Analgesic consumption (paracetamol and tramadol) after discharge home was recorded each week for 1 month.
- Side effects and complications. All side effects and post-discharge complications were recorded. Any re-admission to the hospital following home discharge and the reason for admission was recorded up to 2 years postoperatively.
- Surgical outcomes. Patients were followed-up for a period of 2 years following THA to assess any complications, such as delayed wound healing, surgical site infection, persistent post-surgical pain (PPSP), hip dislocation, re-operation, or readmission to the hospital. Any deaths were recorded.
- Patient-assessed outcomes. Health-related quality of life (HRQoL) was determined preoperatively and after 7 days and 3 and 6 months following surgery using the European Quality of Life–5 Dimensions (EQ-5D) questionnaire and the 36-Item Short Form Health Survey (SF36) preoperatively and after 3 and 6 months postoperatively. Additionally, hip function was assessed using a standardized and validated questionnaire, the Hip dysfunction and Osteoarthritis Outcome Score (HOOS) preoperatively and after 14 days and 3 and 6 months following surgery.

- The EQ-5D questionnaire is a descriptive system of HRQoL states consisting of five dimensions (mobility, self-care, usual activities, pain/discomfort, anxiety/depression), each of which can elicit one of three responses. A weighted average is constructed that varies from 1.0 (completely healthy) to 0 (dead). The Swedish version is derived from the original British version.
- The SF-36 is a multi-purpose, short-form health survey with 36 questions. It yields an 8-scale profile of functional health and well-being scores as well as psychometrically based physical [physical component score (PCS)] and mental [mental component score (MCS)] health summary measures and a preference-based health utility index. A score of zero is equivalent to maximum disability and a score of 100 is equivalent to no disability.
- The HOOS evaluates symptoms and functional limitations related to the hip. It consists of 39 items, assessing five separate patient-relevant dimensions: pain (P) (nine items); symptoms (S), including stiffness and range of motion (five items); activity limitations-daily living (A) (17 items); sport and recreation function (SP) (four items); hip-related quality of life (Q) (four items). To answer each question, five Likert-boxes are used (no, mild, moderate, severe, extreme). All items are scored from zero to four, and each of the five subscales is calculated as the sum of the items included. To enhance the interpretation, HOOS is transformed into a 0–100 (best to worst) scale.

### Statistics

Continuous variables were summarized using the mean ± standard deviation or the median with range, and categorical variables as percentages. The chi-squared test, or Fisher exact test when appropriate, was used to compare study groups for categorical data, with measures of association being the odds ratio with 95% confidence intervals. In view of the study setting with repeated measurements on subjects, we applied a mixed model with unstructured correlation structure to evaluate outcome variables EQ-5D, SF-36 (PCS and MCS), and different HOOS scores. Study group, time, and statistical interaction (group × time) were independent variables in the model as well as preoperative measurement of the outcome, the latter to adjust for mean differences in outcome between study groups at baseline. All independent variables were evaluated on a categorical scale, with the exception of preoperative outcome measurement, which was evaluated on a continuous scale. A statistically significant interaction indicates different mean differences of an outcome between study groups at different postoperative time-points. Analgesic consumption during the period 8–14 days following surgery and NRS pain scores were analyzed using the Mann–Whitney *U* test. HOOS markers were non-normally distributed and therefore evaluated after logarithmic transformations. *P* values of < 0.05 were considered to be statistically significant. All statistical analyses were performed using SPSS version 22 (IBM Corp., Armonk, NY) or STATA release 11 (StataCorp LP, College Station, TX).

## Results

**Fig. 1:**
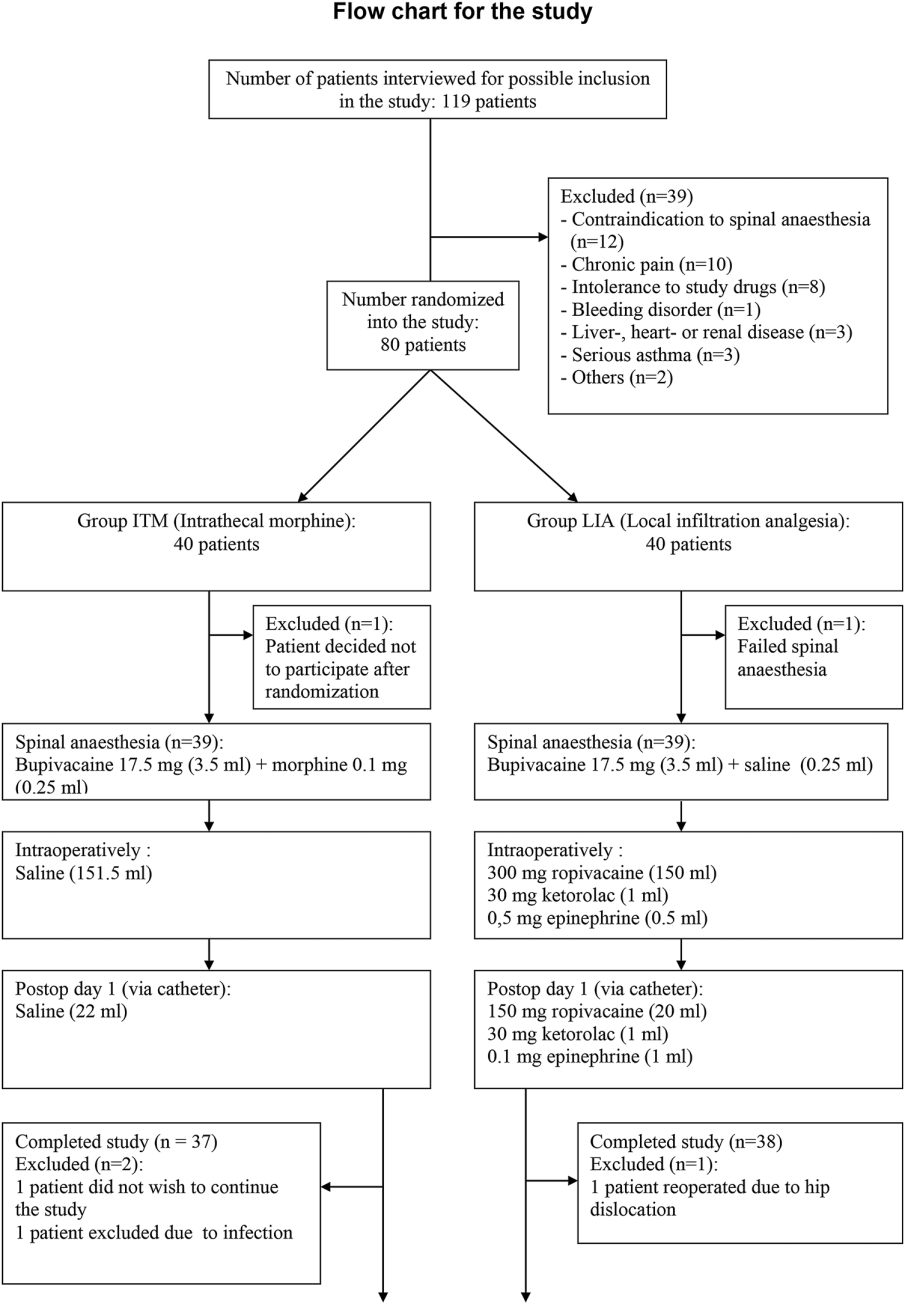
Flow chart for patient inclusion/exclusion and study design. LIA Local infiltration analgesia, ITM intrathecal morphine

A total of 80 patients were randomized, but two patients were subsequently excluded from the analysis, one in each group: one patient decided not to continue the study after randomization (Group ITM) and the other patient left the study because the spinal anesthetic did not achieve adequate surgical analgesia (Group LIA) (this patient subsequently received general anesthesia). Three additional patients were excluded from the analysis of the post-discharge follow-up: two patients in Group ITM (one with suspected postoperative infection and the other patient did not wish to continue with the study) and one patient in Group LIA (postoperative re-operation due to loosening of the cup) (Fig. 1). The age (66 ± 9 and 68 ± 8), weight (83 ± 23 and 86 ± 20), and ASA I/II physical status (12/25 and 14/24) were comparable between patients in Groups ITM and LIA, respectively.

**Fig. 2:**
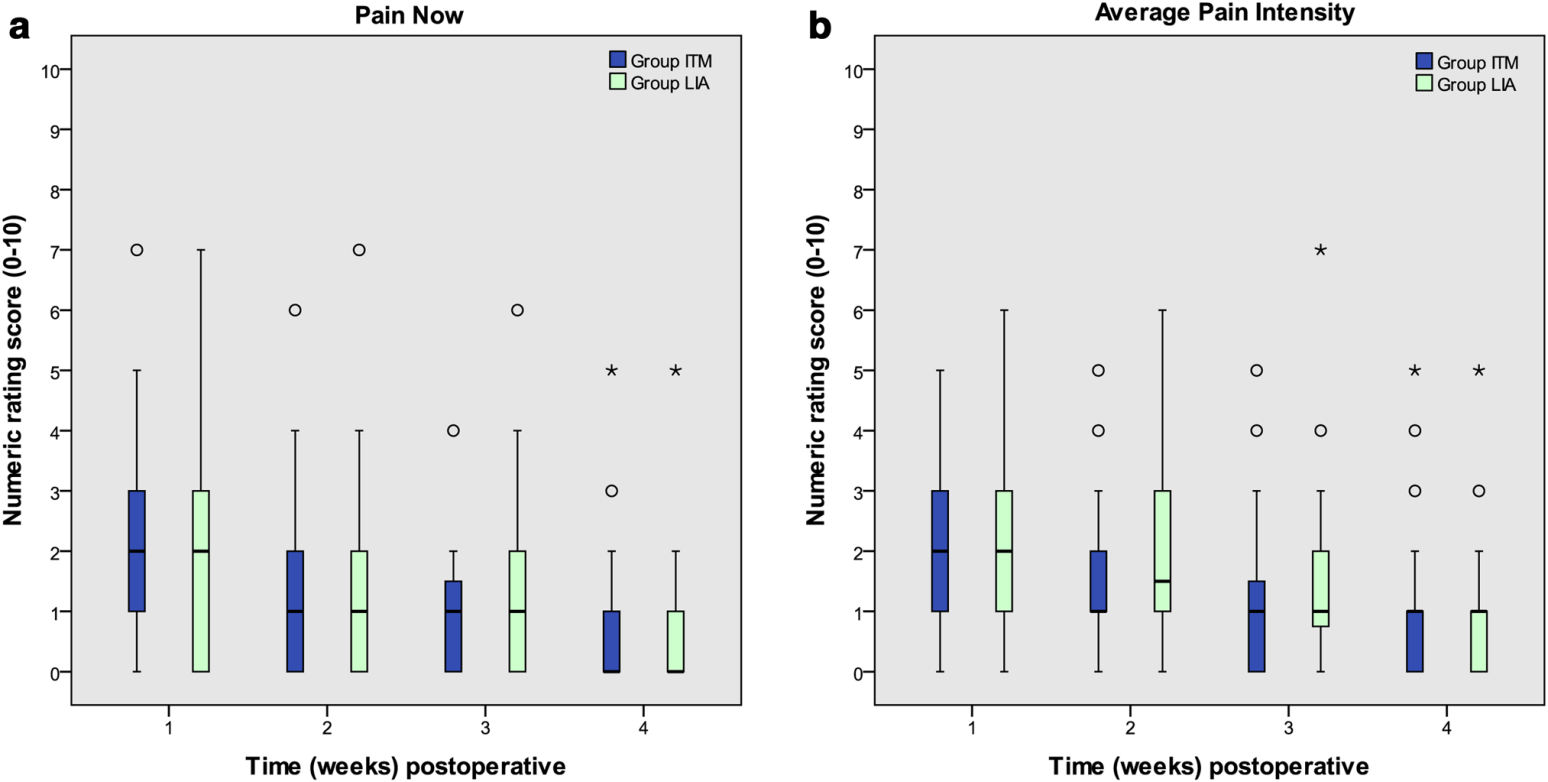
Pain intensity now (**a**) and pain intensity average (**b**) is shown as a box plot during 1–4 weeks following surgery. Circles and asterisks represent outliers

No statistical differences were found in the NRS (pain now and average pain during the preceding week) between the groups during postoperative weeks 1–4 (Fig. 2a, b). In general, average pain scores as assessed with the NRS were low (<3) in both groups at all measurement points.

No significant differences were found in rescue analgesic consumption between Group ITM and Group LIA during postoperative days 8–14 [median (range); paracetamol: 15 (range 0–34) vs. 14 (0–28) g, respectively (*p* = 0.417); tramadol: 250 (0–2100) mg vs. 0 (0–2100) mg, respectively (*p* = 0.314)]. The incidence of persistent post-surgical pain (NRS > 3 or HOOS > 30) in the two groups is shown in Table 1. No statistically significant differences were found in the incidence of PPSP between the groups at either 3 or 6 months (*p* = 0.115). Two patients (6%) in group ITM had pain (NRS > 0) during walking at 6 months following surgery.

**Table 1:** Incidence of persistent post-surgical pain or pain as assessed by the Hip Dysfunction and Osteoarthritis Outcome Score

Pain assessment instruments	3 months postoperative				6 months postoperative			
	Group ITM	Group LIA	LIA vs. ITM		Group ITM	Group LIA	LIA vs. ITM	
			OR (95% CI)	*p* value			OR (95% CI)	*p* value
HOOS	*n* = 36	*n* = 37			*n* = 37	*n* = 36		
HOOS-assessed persistent pain > 30	5 (14%)	10 (27%)	2.3 (0.6–9.6)	0.165	6 (16%)	4 (11%)	0.6 (0.1–3.0)	0.736
NRS	*n* = 35	*n* = 37			*n* = 35	*n* = 37		
>3 (persistent post-surgical pain)	0 (0%)	4 (11%)	NE	0.115	2 (6%)	0 (0%)	NE	0.233

Data are presented as the number of patients (percentage in parenthesis) with persistent post-surgical pain measured using The Hip Dysfunction and Osteoarthritis Outcome Score (HOOS) (>30) and Numeric Rating Score (NRS) (>3) at 3 and 6 months after surgery is presented

* ITM* Intrathecal morphine, *LIA* local infiltration analgesia, *OR* odds ratio, *NE* not estimable, *CI* confidence interval. Please see text for details

HOOS is transformed into: 0 (best score) and 100 (worst score). NRS: 0 = no pain, 10 = worst imaginable pain

One patient in the LIA group had a complete luxation of the hip joint on postoperative day 35 and one patient had sub-luxation on postoperative day 15. Two patients in the ITM group were re-admitted because of pain and difficulty in mobilization on postoperative day 10 and after 4.5 months, respectively, and one had sub-luxation at postoperative day 14. Additionally, one patient in the ITM group had deep vein thrombosis after 4.5 months, and one in Group LIA was admitted to the hospital due to a suspected allergic reaction to diclofenac after 3 months. No patient in either group had surgical site infection. Other significant side effects during the 2-year follow-up are presented in Table 2.

**Table 2:** Postoperative complications

Postoperative complications	1 week–6 months postoperative surgery			6–24 months postoperative		
	Group ITM (*n* = 37)^a^	Group LIA (*n* = 38)^b^	*p* value	Group ITM (*n* = 37)	Group LIA (*n* = 37)	*p* value
Luxation of hip joint	1	2	1.000	0	1^e^	1.000
Pain	2	0	0.240	0	1	1.000
Deep vein thrombosis	1	0	0.493			
Allergic reaction to oral diclofenac	0	1	1.000			
MACE						
AV-block III (pacemaker)				0	1	1.000
Syncope (arrhythmias)				1	0	1.000
Ascending aorta aneurysm and CABG (elective surgery)				0	1	1.000
Death	0	1^c^	1.000	1^d^	0	1.000

Data in the table are presented as the number of patients

*MACE* major adverse cardiac events,* AV* Atrioventricular,* CABG* coronary artery bypass grafting

^a^ One patient was excluded 2 days after randomization due to suspected systemic infection

^b^One patient was excluded after 7 days due to hip dislocation

^c^Pneumonia, 5 months after surgery

^d^Lung cancer, 22 months after surgery

^e^Same patient had luxation on several occasions

There was an overall improvement with time in the HRQoL as assessed using the EQ-5D values (*p* < 0.001), with a significant interaction (group × time) (*p* = 0.036); the latter indicated somewhat higher scores (= better health) in Group LIA (Table 3). There was an overall improvement in the summary measure of the physical component of the SF-36 (PCS) over time (*p* < 0.001) (Table 3), but no interaction was found between group × time. No difference in the MCS of the SF-36 was found over time, and no group × time interaction was seen in the MCS (Table 3).

**Table 3:** Health-related quality of life

Health-related quality of life instruments	Preoperative		7 days postoperative			3 months postoperative			6 months postoperative			Overall postoperative *p* value		
												Group	Time	Group × time
	Group ITM (*n* = 37)	Group LIA (*n* = 38)	Group ITM (*n* = 37)	Group LIA (*n* = 38)	*p* value	Group ITM (*n* = 36)	Group LIA (*n* = 37)	*p* value	Group ITM (*n* = 36)	Group LIA (*n* = 35)	*p* value			
EQ-5D	0.53 ± 0.28	0.55 ± 0.26	0.53 ± 0.22	0.63 ± 0.24	0.069	0.84 ± 0.18	0.81 ± 0.21	0.482	0.85 ± 0.21	0.88 ± 0.18	0.567	0.418	<0.001	0.036
SF36^a^														
PCS	26.2 ± 8.0	28.3 ± 7.9				41.3 ± 10.0	39.8 ± 10.3	0.291	45.0 ± 9.4	46.7 ± 11.2	0.678	0.735	<0.001	0.059
MCS	49.2 ± 11.9	51.7 ± 11.8				54.2 ± 8.9	54.6 ± 10.0	0.758	54.5 ± 8.2	53.5 ± 9.4	0.356	0.517	0.764	0.449

Data in table are presented as the mean ± standard deviation (SD). Statistically evaluated with mixed model adjusted for preoperative measurements. See section Statistics for detailed information

EQ-5D European Quality of Life–5 dimensions, *SF36* 36-Item Short Form Health Survey, *PCS* SF36—Physical Component Summary score, *MCS* SF36—Mental Component Summary score. For both the PCS and MCS, a score of zero is equivalent to maximum disability and a score of 100 is equivalent to no disability. For the EQ-5D, 1.0 = completely healthy and 0 = dead. In both scales, the higher the score, the better the quality of life

^a^Only 31 patients completed the SF36 in the LIA group at 6 months

A significant improvement was found over time in all components of HOOS (pain, symptoms including stiffness and range of motion, activity limitation in daily living and hip-related quality of life) (*p* < 0.001), with the exception of the sport and recreation function (Table 4). A significant overall interaction (group × time) was seen only in HOOS-Pain (*p* = 0.004), with somewhat lower HOOS-Pain in Group ITM, specifically at 3 months, but there was no statistical difference between the groups.

**Table 4:** Hip Dysfunction and Osteoarthritis Outcome Score

HOOS^a^	Preoperative		14 days postoperative			3 months postoperative			6 months postoperative			Overall postoperative *p* value		
												Group	Time	Group × time
	ITM (*n* = 37)	LIA (*n* = 38)	ITM (*n* = 37)	LIA (*n* = 37)	*p* value	ITM (*n* = 36)	LIA (*n* = 37)	*p* value	ITM (*n* = 37)	LIA (*n* = 36)	*p* value			
HOOS-Pain	59.2 ± 13.0	60.4 ± 15.1	27.1 ± 18.3	27.5 ± 17.4	0.901	12.3 ± 16.2	18.5 ± 18.7	0.098	11.9 ± 13.6	11.5 ± 13.6	0.694	0.610	<0.001	0.004
HOOS-Symptoms	64.7 ± 10.9	60.4 ± 18.1	30.3 ± 17.4	28.8 ± 17.2	0.943	16.2 ± 12.0	24.6 ± 20.3	0.510	17.3 ± 17.7	16.7 ± 14.3	0.495	0.530	<0.001	0.787
HOOS-ADL	57.7 ± 12.5	57.2 ± 18.4	32.7 ± 18.2	30.5 ± 17.0	0.449	19.6 ± 18.6	23.8 ± 18.4	0.579	17.9 ± 17.3	15.5 ± 15.0	0.447	0.698	<0.001	0.075
HOOS-SR	84.6 ± 13.1	78.8 ± 15.7	42.1 ± 33.6	47.5 ± 31.5	0.748	41.8 ± 27.7	43.1 ± 29.0	0.968	35.3 ± 26.4	34.5 ± 26.0	0.835	0.796	0.075	0.944
HOOS-QOL	78.9 ± 9.7	75.0 ± 12.9	54.2 ± 27.2	50.0 ± 19.1	0.686	36.8 ± 24.1	36.3 ± 25.4	0.836	29.4 ± 21.4	24.5 ± 19.4	0.769	0.908	<0.001	0.836

Results of HOOS questionnaire are shown as mean ± SD. Statistically evaluated with mixed model adjusted for preoperative measurements, HOOS evaluated on log scale due to non-normal distributions. See section Statistics for detailed information

^a^The HOOS consists of 39 items, assessing five separate patient-relevant dimensions: Pain, Symptoms (including stiffness and range of motion), ADL (Activity limitations-Daily Living), SR (Sport and Recreation function), QOL (Hip-Related Quality of Life). HOOS is transformed into a 0–100 scale, where 0 = best and 100 = worst outcome score

## Discussion

In this study, we found no differences in pain intensity or analgesic consumption after home discharge between patients receiving LIA and those receiving ITM after THA. Functional recovery was similar between the two treatment groups for up to 6 months after surgery. No difference in PPSP was found between the groups at 3–6 months following surgery, and the incidence of post-surgical complications was similar between the groups up to 2 years later. A significant improvement in HRQoL was found using the EQ-5D tool but not with the SF-36 in the LIA group, postoperatively. The small difference seen between the groups in these endpoints has minimal clinical importance, and we therefore believe that LIA is a safe technique when used for the management of postoperative pain following THA.

We used a specific instrument for the assessment of hip function, i.e., the HOOS. This questionnaire has been used in patients with hip diseases with or without arthritis [13]. It is used to assess the impact of treatment of hip diseases as well as to describe the consequence of exposure of a population of patients to a treatment or management strategy. The Western Ontario and McMaster Universities Arthritis Index score has also been used in this setting, but we found that the HOOS is a simple and easily understood questionnaire, and it has been widely used in the Swedish population. In the present study, patients in both groups showed a steady improvement over time in all components of the HOOS score, with the exception of the dimension sports and recreation. All patients were back to normal function and activities related to the hip after 6 months. No inter-group differences were found, except in the pain component of HOOS for which a significantly greater number of patients in the LIA group had a pain score of > 30. This aspect is discussed in greater detail in the following text.

Orthopedic surgeons have often been concerned about the use of NSAIDs in patients undergoing orthopedic procedures [14]. Since prostaglandins play an important role in the regulation of osteoblast and osteoclast formation, inhibition of prostaglandin production due to the use of NSAIDs may retard bone formation [11] or lead to renal insufficiency, specifically in elderly patients. Therefore, NSAIDs may be expected to have significant consequences when used in several clinical situations where bone formation or remodeling is an important factor in healing, as during bone fractures [15] or non-cemented hip prostheses. However, it is important that the advantages of NSAIDs for pain relief should be weighed against the possible risk for delayed healing, and caution should be exercised during the first two postoperative weeks, specifically after fracture surgery. In a study on rats, celecoxib therapy was found to worsen the mechanical properties of callus formation during the early phase of fracture repair [16], possibly due to inhibition of angiogenesis [17]. However, no clinical evidence of prosthesis migration or differences in pain scores, range of motion, and subjective outcome were found after 2 years when comparing celecoxib with placebo in patients undergoing total knee replacement [18]. In the present study, two patients in the LIA group (5.4%) who were administered peri-articular injections of ketorolac experienced prosthetic dislocation during the 2-year post-discharge follow-up. In contrast, in a multi-centre study from France that involved > 2000 primary hip arthroplasties, 10.4% of all patients who underwent revision arthroplasty had hip dislocation [2]. This is similar to the results obtained from the Swedish arthroplasty register where 8.7% of patients underwent revision arthroplasty due to dislocation. In our study, a total of three patients had hip dislocation (4%), with no inter-group difference.

The incidence of PPSP, as measured by NRS, and rescue pain requirement was similar between our two study groups during the 6-month follow-up. We used pain intensity exceeding 3 on the NRS or 30 on the pain component of HOOS when defining PPSP. There is no clear international definition of pain intensity when assessing PPSP and therefore the reported incidence varies considerably (27–38%) in patients undergoing THA [1]. The low incidence of PPSP in our study (<10% on NRS) could be due to our use of a more strict definition, i.e., pain intensity > 3 on NRS or > 30 on the pain component of HOOS. PPSP has been a matter of concern, and several studies have recently highlighted this problem [1, 7]. Prior to the start of our study, we were concerned that the use of NSAIDs in the LIA group may manifest as PPSP syndrome due to delayed healing. However, PPSP does not seem to have been a clinical problem in the small group of patients we studied over 2 years. Thus, despite theoretical arguments as well as results from animal studies suggesting that NSAIDs should not be administered during orthopaedic surgery, we found that two doses of ketorolac given peri-articularly had no clinically relevant adverse effects while offering good postoperative analgesia.

Local anaesthetics have been used for the management of postoperative pain and have been injected in different tissue planes with good effect [19]. Although a single dose of LA appears to have only a short effect-duration [20], the use of catheters and the intermittent injection of LA peri-neurally, subcutaneously, intra-fascially, as well as intra-abdominally have prolonged the duration of analgesia [9, 21]. Despite the fear of systemic absorption when using large doses of LA, as during LIA, toxic plasma concentrations have not been measured, and only rarely have adverse effects been described [22].

We used two different questionnaires to assess HRQoL. The EQ-5D is a standardized method to assess quality of life in five dimensions. The questionnaire is well validated, but it is non-specific and can therefore be used in different situations [23]. The average value on the EQ-5D provides an assessment of patient-assessed health and quality of life, with high scores suggesting good health. We found that there was a slight deterioration in the scores on the EQ-5D at 7 days postoperatively compared to preoperative values, with higher scores in the LIA group. Thereafter, there was a steady improvement, and at 6 months, patients had reached a high quality of life suggesting a significant improvement in perceived health, compared to preoperative values. Similarly, we also found an improvement over time in patient-assessed HRQoL using the physical component score (PCS) of the SF-36 in both groups, but not in the mental component score (MCS), suggesting that physical improvement in health may not necessarily translate into mental improvement.

### Study limitations

This study was a follow-up of post-discharge data collected from a larger study performed to investigate the LIA technique for postoperative pain management. Therefore, power calculations were done on the basis of postoperative pain and not post-discharge events or quality of life. The study is consequently underpowered to detect uncommon hip complications following THA. For example, assuming that the incidence of hip dislocation requiring revision arthroplasties is 10%, it would require about 400 patients to see a doubling of the incidence in the LIA group. On the other hand, in our series of 80 patients, and assuming an overall incidence of 10%, we should have seen at least eight patients with major complications, which we did not. Additionally, if hip complications requiring re-operation were common when using NSAID in the LIA technique, we would have detected these over the last 10 years since we started using the technique because > 1000 patients have today been exposed to LIA during total hip and knee arthroplasties at our hospital. Therefore, and despite the small number of patients recruited into this study, we believe that the risk for worsened hip function, persistent pain, or delayed-healing appears to be minor and that the use of LIA did not appear to worsen these outcomes.

## Conclusions

Functional recovery after THA did not differ between patients receiving LIA and those receiving ITM at 6 months following surgery. Post-discharge pain, analgesic consumption, and PPSP were similar between the groups. HRQoL at 6 months and the incidence of adverse events up to 2 years following the surgery were also similar between the groups. LIA would appear to be a good alternative to ITM for postoperative pain management following THA and to be associated with mild post-discharge pain, satisfactory functional recovery, a good HRQoL, a low risk for PPSP at 6 months and a low incidence of adverse events up to 2 years after surgery.
